# Improving sudden cardiac death risk stratification in hypertrophic cardiomyopathy using established clinical variables and genetic information

**DOI:** 10.1007/s00392-023-02310-4

**Published:** 2023-10-04

**Authors:** Ali Amr, Jan Koelemen, Christoph Reich, Farbod Sedaghat-Hamedani, Elham Kayvanpour, Jan Haas, Karen Frese, David Lehmann, Hugo A. Katus, Norbert Frey, Benjamin Meder

**Affiliations:** 1 Institute for Cardiomyopathies & Center for Cardiogenetics, Department of Medicine III, University of Heidelberg, Im Neuenheimer Feld 410, 69120 Heidelberg, Germany; 2 DZHK (German Centre for Cardiovascular Research), Standort Heidelberg/Mannheim, 69120 Heidelberg, Germany; 3 Stanford Genome Technology Center, Stanford University School of Medicine, Palo Alto, CA 94305 USA

**Keywords:** Hypertrophic cardiomyopathy, Sudden cardiac death, Risk stratification, Genetic testing, ICD implantation

## Abstract

**Background and aims:**

The cardiac societies of Europe and the United States have established different risk models for preventing sudden cardiac death (SCD) in hypertrophic cardiomyopathy (HCM). The aim of this study is to validate current SCD risk prediction methods in a German HCM cohort and to improve them by the addition of genotype information.

**Methods:**

HCM patients without prior SCD or equivalent arrhythmic events ≥ 18 years of age were enrolled in an expert cardiomyopathy center in Germany. The primary endpoint was defined as SCD/-equivalent within 5 years of baseline evaluation. 5-year SCD-risk estimates and recommendations for ICD implantations, as defined by the ESC and AHA/ACC guidelines, were analyzed. Multivariate cox proportional hazards analyses were integrated with genetic findings as additive SCD risk.

**Results:**

283 patients were included and followed for in median 5.77 years (2.92; 8.85). A disease-causing variant was found in 138 (49%) patients. 14 (5%) patients reached the SCD endpoint (5-year incidence 4.9%). Kaplan–Meier survival analysis shows significantly lower overall SCD event-free survival for patients with an identified disease-causing variant (p < 0.05). The ESC HCM Risk-SCD model showed an area-under-the-curve (AUC) of 0.74 (95% CI 0.68–0.79; p < 0.0001) with a sensitivity of 0.29 (95% CI 0.08–0.58) and specificity of 0.83 (95% CI 0.78–0.88) for a risk estimate ≥ 6%/5-years. By comparison, the AHA/ACC HCM SCD risk stratification model showed an AUC of 0.70 (95% CI 0.65–0.76; p = 0.003) with a sensitivity of 0.93 (95% CI, 0.66–0.998) and specificity of 0.28 (95% CI 0.23–0.34) at the respective cut-off. The modified SCD Risk Score with genetic information yielded an AUC of 0.76 (95% CI 0.71–0.81; p < 0.0001) with a sensitivity of 0.86 (95% CI 0.57–0.98) and specificity of 0.69 (95% CI 0.63–0.74). The number-needed-to-treat (NNT) to prevent 1 SCD event by prophylactic ICD-implantation is 13 for the ESC model, 28 for AHA/ACC and 9 for the modified Genotype-model.

**Conclusion:**

This study confirms the performance of current risk models in clinical decision making. The integration of genetic findings into current SCD risk stratification methods seem feasible and can add in decision making, especially in borderline risk-groups. A subgroup of patients with high SCD risk remains unidentified by current risk scores.

**Graphical abstract:**

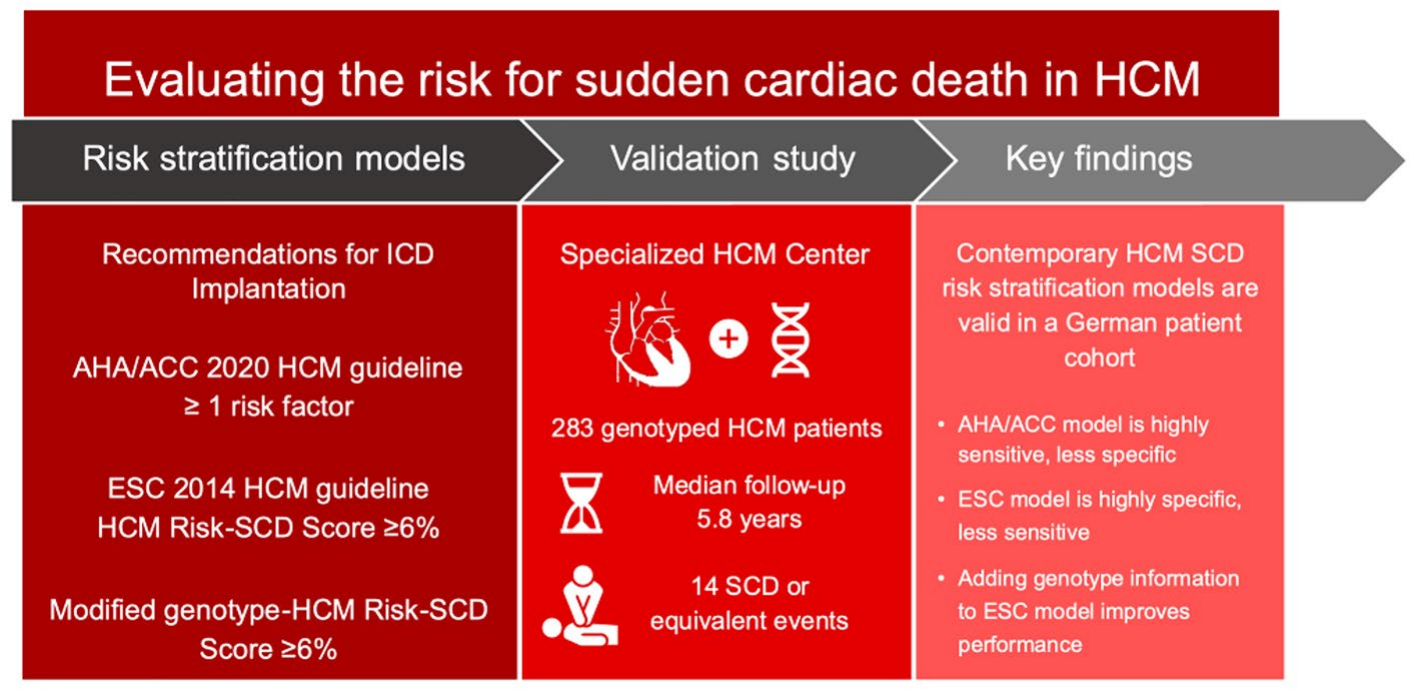

## Introduction

Hypertrophic cardiomyopathy (HCM) is the most prevalent genetic myocardial disease [1, 2, 22]. It can be morphologically defined by non-secondary, left ventricular hypertrophy. Histopathological characteristics of HCM are hypertrophied myocytes with cellular disarray and marked interstitial fibrosis [10]. The most common cause of HCM is the presence of disease-associated variants in genes coding for sarcomeric proteins [1, 20]. The wide availability of cardiac imaging capabilities and the increasing awareness of HCM has enabled clinicians to successfully and reliably identify more patients suffering from HCM at earlier stages of the disease [12, 15, 21, 23] and with different disease manifestations, e.g. the dynamic obstruction of blood-flow in the left ventricular outflow tract (LVOT) [4, 10, 21].

HCM remains one of the main causes of sudden cardiac death (SCD) in young patients even though the majority of the patients present mild symptoms at diagnosis. Previous studies and registers describing the natural history of HCM have reported an increased risk of SCD in HCM patients in comparison to the general population. Several risk factors have been identified over the course of the last 50 years that are associated with an increased risk of SCD. Although SCD is a tragic and devastating complication, the absolute number of events that is reported in HCM patient registries is relatively low and ranges at around 1.1% per year [14, 17, 24]. An adequate SCD risk assessment for all patients with HCM is essential and includes comprehensive clinical and family history, Holter-monitoring, ECG, cardiac imaging and exercise testing [4, 16, 26]. Careful consideration is warranted when identifying patients that qualify for a primary prophylactic ICD implantation. On one hand, the emphasis certainly relies on reaching a higher sensitivity to avoid missing patients with a higher risk of SCD. On the other hand, increasing the sensitivity using the currently available clinical tools will put more patients at risk of complications of device therapy without having a need.

An underlying disease-causing mutation can be identified in up to 60 percent of the patients with HCM [1, 4, 20]. In accordance with ESC and AHA guidelines, genetic testing should be discussed and evaluated for each patient suffering from HCM [1, 4, 6, 13, 27]. The majority of gene variants can be found in cardiac myosin-binding protein C (*MYBPC3*) gene and the myosin heavy chain (*MYH7*) gene [2, 20] with sarcomere mutations being associated with worse outcome [3, 5, 7, 8, 11, 29]. Still, the use of this genetic information in clinical routine is still sparse in many countries.

The ESC HCM guidelines recommend the incorporation of the 5-year SCD Risk Score Calculator (ESC RSC) in the decision making (which in effect relatively favors specificity). The 5-year Risk Score Calculator model has been developed through a multicenter study of 3675 patients [26]. Using clinical parameters only, the model estimates the individual 5-year SCD risk and divides the patients into three categories: patients with low (< 4% risk in 5 years), intermediate and high risk (> 6% per 5 years) [4, 26]. Current guidelines recommend primary prophylactic ICD-implantation considering the individual SCD risk for each patient with the threshold being 5-year SCD risk ≥ 6% for the ESC model (Class IIb recommendation) or the presence of ≥ 1 risk factor for the 2020 AHA/ACC approach (Class IIa/b recommendation) [4, 27]. Genetic results are not integrated into the SCD risk stratification, although the incidence of malignant arrhythmia is reported to be higher in patients with variants in genes encoding sarcomeric proteins [17, 18, 29, 30]. Patients carrying a disease-causing variant (genotype-positive) are reported to have a worse outcome than patients without a disease-causing mutation (genotype-negative) [17, 18, 29, 30]. The relatively high costs of genetic sequencing and low accessibility in many countries has prohibited integration in the risk models.

The aim of this study is twofold. Firstly, we aim to validate the ESC RSC and the AHA HCM risk stratification guidelines in a German HCM patient population in a longitudinal study. Secondly, to investigate the benefit of integrating genetic findings into the ESC 5-year risk model.

## Methods

### Study design

This project is planned as a single-center, longitudinal study of patients with primary hypertrophic cardiomyopathy. Clinical examinations, diagnostic procedures and follow-up were performed in adherence to hospital guidelines. This study is in line with the principles of the Helsinki Declaration. The written study proposal and protocol have been formally submitted and accepted by the ethics committee of the Medical Faculty of the University of Heidelberg.

### Study population

Index patients with primary HCM that visited the University Hospital Heidelberg between 2001 and 2017 were consequently recruited. HCM was defined by unexplained maximum left ventricular wall thickness ≥ 15 mm or in accordance with published criteria for the disease diagnosis in relatives of patients with HCM [4, 25, 27]. Secondary causes for the hypertrophy, like uncontrolled hypertension, valvular disease, inflammatory diseases, amyloidosis, as well as syndromic and metabolic etiologies were excluded.

All patients underwent comprehensive clinical evaluation. The initial clinical assessment included a comprehensive family history, ECG, echocardiography, laboratory parameters and ergometry. When needed, further phenotyping was performed to exclude secondary causes using cardiac MRI, LV Biopsy and/or coronary angiography. All subsequent clinical visitations were documented. Patients that did not return for clinical follow-up visitations were contacted by mail and telephone.

The ESC RSC was calculated for all patients at first presentation: age, positive family history (1st degree relative with SCD < 40 years or confirmed HCM), maximum left ventricular wall thickness (mm), maximum left ventricular outflow tract gradient at rest and under Valsalva maneuvers (mmHg), non-sustained ventricular tachycardias (< 30 s, > 120 b/min) at or before first presentation and syncope of unclear origin at or before first presentation.

The score was calculated using the originally published formula:

$${\text{P}}_{{\text{SCD at 5 years}}} = {1} - 0.{998}^{{{\text{exp }}({\text{Prognostic Index}})}} ,$$

Prognostic Index = 0.15939858*(Maximal wall thickness (mm)) – 0.00294271*(Maximal wall thickness)^2^ (mm^2^) + 0.0259082*(Left atrial diameter (mm)) + 0.00446131*(Maximal left ventricular outflow tract gradient (mmHg)) + 0.4583082*(Family history SCD) + 0.82639195*(Non-sustained ventricular tachycardia (NSVT)) + 0.71650361*(Unexplained syncope)—0.01799934*(Age at clinical evaluation (years)).

SCD risk in the study population was also assessed using AHA/ACC guideline recommendations for SCD risk stratification [27]. Established risk factors are family history of sudden death from HCM, massive left ventricular hypertrophy ≥ 30mm, unexplained syncope, HCM with systolic LV-dysfunction (LV-EF < 50%), LV apical aneurysm, Extensive LGE on CMR imaging, NSVT on ambulatory monitor) [27].

### Endpoint definition

The primary endpoint of the study was sudden cardiac death or an equivalent event after the initial presentation. SCD-equivalent events include appropriate ICD shock therapy or adequate anti-tachycardia pacing (ATP) in patients with ICD (as primary prevention), as well as documented hemodynamically relevant persistent ventricular tachycardias > 30s, ventricular flutter or fibrillation. The endpoints were documented through follow-up visitations, discharge letters from external hospitals, via postal mail and telephone queries.

### Integration of genetic findings in SCD risk stratification

A literature search was performed using PubMed for studies on genotype–phenotype associations in HCM patients with available survival time analysis. Two studies were identified that provided information on the hazard ratio for SCD [18, 30]. Patients that underwent genetic testing were then divided into two groups depending on the results of the genetic testing (genotype positive vs. genotype negative). A genetic result as a parameter was then added to the original ESC Risk score calculator using cox proportional hazards model as follows:

$${\text{P}}_{{\text{SCD at 5 years}}} = {1} - \, 0.{998}^{{{\text{exp }}({\text{Prognostic Index}})}} ,$$

Prognostic Index = 0.15939858*(Maximal wall thickness (mm)) – 0.00294271*(Maximal wall thickness)^2^ (mm^2^) + 0.0259082*(Left atrial diameter (mm)) + 0.00446131*(Maximal left ventricular outflow tract gradient (mmHg)) + 0.4583082*(Family history SCD) + 0.82639195*(NSVT) + 0.71650361*(Unexplained syncope)—0.01799934*(Age at clinical evaluation (years)) + 1.0612565 *(Genotype positive^#^).

^#^(Hazard ratio for SCD-endpoint = 2.89; Ln (2.89) = 1.0612565).

### Statistical analysis

A descriptive statistical analysis of the patient population was performed. Categorical and nominal variables were analyzed using Fisher’s exact test or Chi-Square test at baseline and follow-up. Parametric and non-parametric statistical methods were applied for observations that are temporal independent or dependent (time series/non-time series). ROC curve analyses (DeLong) were performed using MedCalc (version 19.6.4) and R (version 4.1.2) to assess the performance of the SCD Risk scores in this patient population. Kaplan–Meier survival graphs were also created for the patient population and subpopulations. The sensitivity, specificity, positive (PPV) and negative predictive value (NPV), accuracy, positive and negative likelihood ratio (LR) and the number-needed-to- treat (NNT) were calculated.

## Results

### Genotype is associated with higher mortality

A final set of in total n = 283 HCM patients who visited the Heidelberg cardiomyopathy out-patient clinic and underwent genetic testing were included in this study. Baseline characteristics of the study cohort are given in Table 1. 67% of the patients were male and the mean age was 50 ± 15.4 years. 36% of the patients reported that one or more first-degree relatives suffered from a cardiomyopathy. The yield of the genetic testing was 49% for the detection of class 4 or 5 variants (likely pathogenic (LP) and pathogenic (P)) according to ACMG criteria [28]. The most common genetic findings were variants in *MYBPC3* and *MYH7* (43%). The majority of the patients were not severely limited by heart failure symptoms, with NYHA class I and II (88%) making up the majority of the patient population. The obstructive form was present in 41% of the patient population at baseline. The mean LVOT gradient in HOCM patients was 57.33 ± 50.35 mmHg. An apical aneurysm was found in 6% of patients. With regards to drug therapy, there were no significant differences between HOCM and HNCM at baseline regarding β-blocker therapy (70% vs 72%; p = 0.63). Patients with HOCM significantly more often received non vasodilating calcium antagonists (22% vs. 7%; p < 0.001). Patients with novel myosin-inhibitors were not presented in the cohort.

**Table 1:** Baseline characteristics of the patient population

Patient characteristics at baseline	Values	Patient characteristics at baseline	Values
Patients, number (total)	283	Atrial fibrillation, number (%)	68 (24)
Female, number (%)	93 (33)	Family history of CMP, number (%)	101 (36)
Male, number (%)	190 (67)	Family history of SCD, number (%)	32 (11)
Phenotype		Syncope prior to visit, number (%)	46 (16)
HNCM, number (%)	167 (59)	nsVT prior to visit, number (%)	66 (23)
HOCM, number (%)	116 (41)	ICD-implantation, number (%)	17 (6)
Genetically tested patients, number (%)	283 (100)	NYHA I, number (%)	137 (48)
Genotype positive, number (%)	138 (49)	NYHA II, number (%)	112 (40)
MYBPC3, number (%)	90 (32)	NYHA III, number (%)	34 (12)
MYH7, number (%)	33 (12)	NYHA IV, number (%)	0 (0)
TNNT2, number (%)	8 (3)	6MWT, mean ± SD, m	513.19 ± 111.74
TNNI3, number (%)	3 (1)	Laboratory results	
TPM1, number (%)	2 (1)	NT-proBNP, median (1Q;3Q), ng/l	572 (204; 1363.5)
Other variants, number (%)	3 (1)	hs-TNT, median (1Q;3Q), pg/l	11 (7; 21)
Genotype negative, number (%)	145 (51)	Echocardiography	
Age at visit, mean ± SD, years	50 ± 15.42	LV ejection fraction, mean ± SD, %	53.70 ± 6.67
Age at diagnosis, mean ± SD, years	45.53 ± 18.15	Max. LA diameter, median (1Q;3Q), mm	43 (37; 47)
Height, mean ± SD, cm	1.73 ± 0.09	Max. LV wall thickness, median (1Q;3Q), mm	18 (15.5; 23)
Weight, mean ± SD, kg	81.99 ± 16.31	Max. LVOT gradient, HNCM-patients, mean ± SD, mmHg	4.9 ± 4.41
BMI, mean ± SD, kg/m2	27.23 ± 4.91	Max. LVOT gradient, HOCM-patients, mean ± SD, mmHg	57.33 ± 50.35
Heart rate, mean ± SD, beats/min	70.09 ± 14.57	Coronary angiography, number (%)	214 (76)
Blood pressure		Cardiac index, mean ± SD, (l/min)/m2	2.7 ± 0.72
Systolic, mean ± SD, mmHg	128.6 ± 20.27	Cardiac MRI, number (%)	220 (78)
Diastolic, mean ± SD, mmHg	78.65 ± 12.1	Late Gadolinium Enhancement, number (%)	126 (45)
Left bundle-branch block, number (%)	28 (10)	Apical aneurysm, number (%)	16 (6)

*SD* denotes standard deviation, *HNCM* hypertrophic non-obstructive cardiomyopathy, *HOCM* hypertrophic obstructive cardiomyopathy, *MYPBC3* myosin binding protein C3, *MYH7* myosin heavy chain 7, *TNNT2* Troponin T2, *TNNI3* Troponin I3, *TPM1* Tropomyosin 1, *BMI* body-mass-index, *CMP* cardiomyopathy, *SCD* sudden cardiac death, *nsVT* non-sustained ventricular tachycardia, *ICD* implantable cardioverter defibrillator, *NYHA* New York heart association, 6MWT 6-min-walking-test, NT-proBNP N-terminal prohormone of brain natriuretic peptide, hs-TNT high-sensitive Troponin T, *LV* left ventricular, *LA* left atrium, *LVOT* left ventricular outflow-tract, *MRI* magnetic resonance imaging

**Fig. 1:**
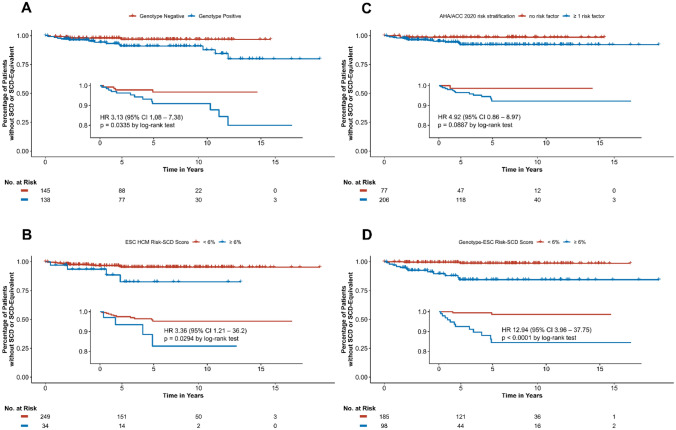
Kaplan-Maier survival curves of primary endpoint. The inset shows the same data on an expanded y-axis. Panel **A** shows survival analysis depending on genotype. Survival is significantly reduced in the genotype positive patient group (Hazard ratio (HR) 3.13, 95% confidence interval (CI) 1.08–7.38; p = 0.0335). Panel **B** shows survival analysis based on the ESC HCM-Risk SCD Calculator score. Threshold was chosen according to current guidelines at 6% 5-year risk value. Survival is significantly reduced in patients with a score ≥ 6% (HR 3.36, 95% CI 1.21–36.2; p = 0.0294). Panel  **C** shows survival analysis based on the AHA/ACC 2020 risk stratification model. Threshold was chosen according to current guidelines by proof of  ≥ 1 SCD-related risk factor. Survival is non-significantly reduced in probands fulfilling ≥ 1 risk factors (HR 4.92, 95% CI 0.86–8.97; p = 0.0887). Panel  **D** shows survival analysis based on the Genotype Risk-SCD Calculator score. Threshold was chosen at 6% 5-year risk value analogue to original ESC HCM Risk-SCD model. Survival is significantly reduced in patients with a score ≥ 6% (HR 12.94, 95% CI 3.96 – 37.75, p < 0.0001). SCD sudden cardiac death, ESC European Society of Cardiology, HCM hypertrophic cardiomyopathy, AHA American Heart Association, ACC American College of Cardiology

The median follow-up time was 5.77 (2.92; 8.85) years. An ICD was implanted in 84 (30%) patients during follow-up. The primary endpoint was reached in 14 patients that suffered SCD or SCD-equivalent event within 5 years after initial evaluation, which is consistent with previously reported rates of SCD in HCM [14, 17, 19, 24]. The majority of patients (n = 10; 71%) that suffered SCD had a pathogenic variant in one of the known HCM disease genes. 6 primary endpoints in patients with HOCM and 1 primary endpoint in the 16 patients with apical aneurysm were observed. A total of n = 34 patients died during the follow-up duration. The most common cause of death was from a non-cardiovascular etiology (74%). Regarding SCD, genotype-positive patients had a significantly lower survival than genotype–negative patients during the follow-up period of 5 years (hazard ratio (HR) 3.13, 95% confidence interval (CI) 1.08–7.38; p = 0.03) (Fig. 1, Panel A).

### Validation of established HCM SCD risk models

**Fig. 2:**
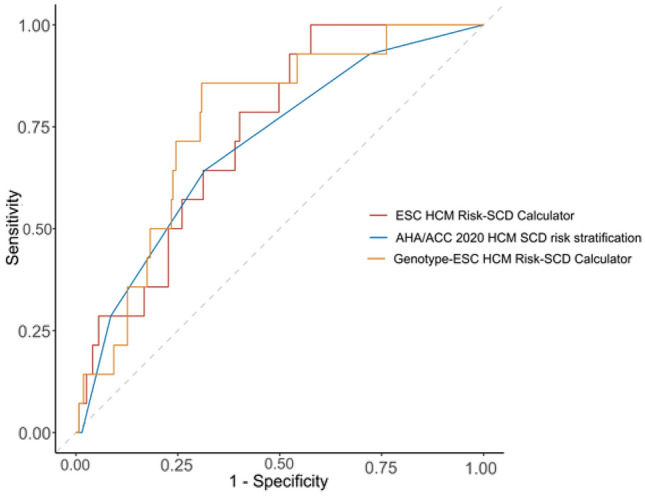
Receiver operating charateristics (ROC) curve analysis for HCM SCD risk stratification models. The area-under-the-curve (AUC) was calculated for each model. Analysis of ESC HCM Risk-SCD Calculator shows an AUC of 0.735 (95% confidence interval (CI) 0.679–0.785; p < 0.001). Analysis of AHA/ACC 2020 HCM SCD risk stratification approach shows an AUC of 0.704 (95% CI 0.647–0.757; p = 0.003). Analysis of modified Genotype-model shows an AUC of 0.76 (95% CI 0.706–0.809; p < 0.001). *SCD* sudden cardiac death, *ESC* European Society of Cardiology, *HCM* hypertrophic cardiomyopathy, *AHA* American Heart Association, *ACC* American College of Cardiology

The ESC SCD Risk model (ESC RSC) was calculated for all patients at baseline to evaluate its performance. A receiver operating characteristics (ROC) evaluation was performed for 5-year follow-up-endpoint and an area under the curve (AUC) of 0.735 (95% CI 0.679–0.785; p < 0.001) was observed. At an ESC-defined cut-off of 6% (“ICD is recommended”), the sensitivity of the ESC RSC was 28.6% (95% CI, 8.4–58.1) and the specificity was 83.3% (95% CI, 78.3–87.5) (Fig. 2). Patients presenting with an ESC RSC of 6% or more had a significantly lower survival probability (HR 3.36, 95% CI 1.21–36.2; p = 0.0294) (Fig. 1, Panel B). The AHA SCD risk stratification was also analyzed in the patient cohort. A statistically non-significant, numerically smaller AUC of 0.704 (95% CI 0.647–0.757; p = 0.003), compared to the ESC RSC, was calculated (Fig. 2). For the threshold of ≥ 1 AHA-defined risk factors (“ICD is reasonable”) the sensitivity of the AHA/ACC guideline recommendations for SCD risk stratification was 92,9% (95% CI 66.1–99.8) and the specificity was 27.9% (95% CI 22.6–33.6) (Fig. 2). The survival analyses of the AHA SCD risk stratification showed no statistical significance in predicting survival probability after categorizing the patients in either 2A or 2B ICD indication groups (HR 4.92, 95% CI 0.86–8.97; p = 0.0887). (Fig. 1, Panel C).

Next, the results of the genotyping were integrated into the ESC RSC as described above, and a new score (Genotype-model) was calculated for all the study subjects. The ROC analyses for this modified Genotype-model showed a significant increase in the AUC from 0.735 to 0.76 (95% CI 0.706–0.809; p < 0.001). Most notably, the sensitivity at the ESC-defined 6% cut-off increased to 85.7% (95% CI 57.2–98.2), while the specificity only slightly decreased to 69.1% (95% CI 63.3–74.6) (Fig. 2). A hazard ratio of HR = 12.94 (95% CI 3.96–37.75) was observed in the survival analysis for patients with a Genotype-model at the cut-off of 6% or more (p < 0.0001) (Fig. 1, Panel D).

The diagnostic performance of all risk stratification models was compared according to the ICD-recommendation cut-off values; *i.e.*, ≥ 6% for the ESC RSC and Genotype-model, or ≥ 1 risk factor for the AHA risk stratification (Table 2). Using this binary approach, the highest sensitivity was observed in the AHA risk stratification approach, correctly detecting 13 from 14 events. However, this approach proved to have the highest NNT (NNT = 28) due to the low specificity in comparison to the ESC RSC (NNT = 13). The Genotype-model achieved the highest precision (PPV = 12.2%), the highest positive likelihood ratio (LR +  = 2.68) and the lowest NNT (NNT = 9), while the original ESC-model had a higher accuracy (85.9% versus 68.9% for genotype-model and 31.5% for AHA model).

**Table 2:** Practical utilization of current guidelines and adjusted Genotype Risk SCD Calculator for guiding primary prophylactic ICD-Implantation

	2014 ESC HCM Guideline ICD recommendation criterion: Score ≥ 6%	Genotype-model based on ESC Risk-SCD-Calculator ICD recommendation criterion: Score ≥ 6%	2020 AHA/ACC HCM Guideline ICD recommendation criterion: ≥ 1 risk factor
Predicted endpoints	4/14	12/14	13/14
Sensitivity (%)	28.6	85.7	92.9
Specificity (%)	83.3	69.1	27.9
Accuracy (%)	85.9	68.9	31.5
NNT	13	9	28
PPV (%)	11.8	12.2	6.3
NPV (%)	96	98.9	98.7
LR+	2.56	2.68	1.29
LR-	0.8	0.21	0.25

The same threshold was used for the Genotype-model

*SCD* Calculator for recommending *ICD* implantation as in the 2014 ESC HCM guidelines. *ESC* denotes European Society of Cardiology, *HCM* hypertrophic cardiomyopathy, *ICD* implantable cardioverter defibrillator, *NNT* number-needed-to-treat, *SCD* sudden cardiac death, AHA American heart association, *ACC* American college of cardiology, PPV positive predictive value, *NPV* negative predictive value, *LR*+ positive likelihood ratio, *LR-* negative likelihood ratio

## Discussion

HCM patients have an increased risk for SCD in comparison to the general population. While HCM remains one of the most common causes of SCD in the young, the absolute risk for HCM individuals is relatively low [14, 17, 19, 24]. By advancing diagnostic standards, more patients with HCM are diagnosed, especially at early stages of the disease. This subsequently increases the difficulty in identifying patients with an appropriate primary prophylactic ICD indication.

The ESC RSC was validated in different external patient populations, mainly from the UK, Spain, Greece and Italy. To our knowledge, this is the first time that the score is validated in a larger German patient population. Besides the European model, we also evaluated the AHA HCM SCD risk stratification approach and compared both methodologies in the same cohort. Both approaches performed well in identifying patients with risk for SCD, achieving an AUC of 0.74 (ESC RSC) and 0.70 (AHA SCD risk stratification). The definition in the cutoffs for ICD indication is chosen differently in both models: The ESC working-group decided to implement a cut-off favoring specificity, which we can find in our cohort with 83.3%, albeit at the cost of a low sensitivity at 28.6% (cut-off of 6%). The AHA SCD risk stratification approach expectedly showed high sensitivity with 92.9%, but at the cost of a low specificity (27.9%) at the AHA cut-off ≥ 1 risk factor. Similar results were shown in previous studies [9, 24, 31]. We evaluated the integration of genetic findings into the risk models to further improve patient stratification. To avoid complexity and increase the ease of use in the daily routine, we relied on the ESC RSC and integrated the new variable: genotype. A higher SCD event rate in genotype-positive patients has been previously reported in literature [18, 29, 30]. While sarcomeric variants in general were shown to result in higher SCD risk, results of contemporary studies do not allow to do a gene or even variant specific prediction. Moreover, the event rate in HCM patients is relatively low, which makes the expansion of the risk score for specific variants and genes currently infeasible. Due to this reason and for simplicity in daily clinical routines, we have chosen to classify the patient population in 2 groups, genotype-positive (LP or P) and genotype-negative patients (Class I-III according to ACMG). The weighting of the genetic factor was calculated analog to the original formula using cox proportional hazards model with data from previously published literature [18, 30]. The modified Genotype-model based on ESC-RSC (with the integration of genetic findings into the formula) seams to improve prediction especially in the patient population identified as low/intermediate risk.

Extreme values and outliers could affect the diagnostic performance of the scores. The clinical decision making regarding the indication of prophylactic ICD implantation can be assumed to be binary, either the cut-off value is reached, and an ICD implantation is warranted, or the cut-off is not reached and an ICD implantation would not be recommended. Using the existing cut-offs from guidelines, we analyzed the performance of the approaches again in a binary approach. Although the original ESC RSC showed a low sensitivity and misclassified 10 patients as low-risk, it correctly identified 96% of the patients that did not have a higher risk of malignant arrhythmia (achieving a very high specificity). The Genotype-model achieved the highest precision, followed by ESC RSC and AHA SCD risk stratification. This is reiterated in the calculation of NNT per risk stratification approach, where the lowest NNT was observed in the Genotype-model. However, it should be noted that the cut-off values for recommending primary prophylactic ICD implantation are determined by the cardiac societies and follow physician consideration, patient expectations, expert opinions and socioeconomic considerations.

A total of 14 events were observed over a follow-up time of 5.77 years. The ESC RSC identified 4 patients correctly, 3 patients were classified as intermediate risk. However, 7 patients were incorrectly classified as low-risk. The modified Genotype-model misclassified 2 cases as low risk. Even with the highly sensitive approach of the AHA recommendations, 1 event would not have been identified in our patient population, but quiet some “unnecessary” ICD implantations would have been performed, judged within the observation period of 5 years. These facts underline the importance of shared decision making.

In summary, current risk stratification approaches are validated in this German population and can aid in the SCD risk stratification in HCM patients. The Genotype-model is not presented as a substitute to the original guidelines, but provides the ability to be integrated in the contemporary care of HCM patients. The derivation of the new model has its limitations: The addition of the genetic score was based on data from an external HCM patient population and might result in changes in model calibration and adaptations of the cut-offs. Furthermore, this is a monocenter study and a model-optimization, fitting, and validation in an external, larger, independent HCM patient population needs to be performed in future studies. Patients treated with novel myosin inhibitors, such as Mavacamten, where not included in this study, as well as children with HCM. Additionally, there remains a small subpopulation of HCM patients that do not present with the typical risk-factors for electrical vulnerability and appear to be misclassified in the current guidelines. Further research is warranted to understand this patient population, especially to identify further SCD risk factors.
